# Decision tree analysis for age estimation in living individuals: integrating cervical and dental radiographic evaluations within a South African population

**DOI:** 10.1007/s00414-023-03154-3

**Published:** 2024-01-02

**Authors:** A. Uys, M. Steyn, D. Botha

**Affiliations:** 1 Department of Anatomy, University of Pretoria, Pretoria, South Africa; 2 Human Variation and Identification Research Unit, School of Anatomical Sciences, Faculty of Health Sciences, University of the Witwatersrand, Johannesburg, South Africa

**Keywords:** Skeletal age, Living individuals, Third molars, Cervical vertebrae, Decision tree analysis

## Abstract

Age estimation in living individuals around the age of 18 years is medico-legally important in undocumented migrant cases and in countries like South Africa where many individuals are devoid of identification documents. Establishing whether an individual is younger than 18 years largely influences the legal procedure that should be followed in dealing with an undocumented individual. The aim of this study was to combine dental third molar and anterior inferior apophysis ossification data for purposes of age estimation, by applying a decision tree analysis. A sample comprising of 871 black South African individuals (*n* = 446 males, 425 = females) with ages ranging between 15 and 24 years was analyzed using panoramic and cephalometric radiographs. Variables related to the left upper and lower third molars and cervical vertebral ring apophysis ossification of C2, C3, and C4 vertebrae analyzed in previous studies were combined in a multifactorial approach. The data were analyzed using a pruned decision tree function for classification. Male and female groups were handled separately as a statistically significant difference was found between the sexes in the original studies. A test sample of 30 individuals was used to determine if this approach could be used with confidence in estimating age of living individuals. The outcomes obtained from the test sample indicated a close correlation between the actual ages (in years and months) and the predicted ages (in years only), demonstrating an average age difference of 0.47 years between the corresponding values. This method showed that the application of decision tree analysis using the combination of third molar and cervical vertebral development is usable and potentially valuable in this application.

## Introduction

Estimating the age of living individuals is important in a number of circumstances which includes criminal investigations, undocumented migrants, refugees and asylum seekers, human trafficking and child labour [1, 2]. In South Africa, as in many other countries, a “child” is defined as a person under 18 years of age [3]. Children below the age of 18 who are suspected of engaging in criminal activities will not undergo the regular adult criminal procedures. Instead, they will be subjected to the child justice process. If an individual is older than 18 years they are no longer classified as a child and bears the full effect of the law [3]. For living individuals, age is therefore very important in this context, especially around the 18-year threshold.

Dental age estimation evaluating third molars (M3) has been widely used and applied to numerous population groups [4–7]. Third molars complete their formation after the onset of puberty and display a long developmental course. The appearance and development of M3 extends across the age interval of 12–22 years. Due to this delayed development, which takes place partly during the phase of steroid-mediated adolescent growth, researchers have observed that males typically reach certain mineralization stages at younger chronological ages compared to females [8]. It should, however, be kept in mind that this may not be true for all populations, as some studies show no significant age difference between male and female mineralization stages [9]. The median age for white South African females to reach stage H, indicating full root closure based on the 10-stage tooth development system by Solari and Abramovitch [4], is 23.01 years. In comparison, males achieve this stage at 22.18 years for mandibular third molars [6]. In a large UK study, M3 development occurred significantly earlier in black individuals compared to white individuals. The study concluded that the age of black individuals will be overestimated if white reference data is used for M3 development [10].

Various methods exist to evaluate M3 root development, and traditionally comprise an 8-stage scoring system [11]. Solari and Abramovitch [4] evaluated M3 using a 10-stage tooth development scoring system by adding F1 and G1 stages to the original Demirjian [11] classification system. The reason for the additional stages was to achieve higher accuracy of the development toward root closure and this has now become a commonly used system [4, 6].

Various forensic age estimation protocols combine dental and other indicators which include skeletal development of the hand and clavicle [12]. The objective of combining age estimation methods is to narrow the predication intervals of an individual and to enhance the precision of forensic age estimations. Combining dental and skeletal indicators are recommended by various studies and policy reports [13–16]. Skeletal development compliments the information regarding the development of M3 to estimate the age of juveniles and young adults [17]. Recently, Uys et al. [18] found that the anterior inferior apophysis ossification stages of cervical vertebrae C2, C3, and C4 can be used as a reliable indicator to determine the likelihood of being 18 years of age. In this study, for both ancestry (South African black and white) and sex groups, the median ages at which stages 0, 1, and 2 were reached were below the 18-year threshold [18]. The method was also applied to Turkish individuals using CT scans and found to be a valuable addition to current age estimation modalities [19].

Currently the most frequently employed statistical multifactorial models are logistic regression or an approach conceived in a Bayesian framework [20–23]. Recently, decision trees have been proposed as an alternative to transition analysis with potentially more accurate (or similar) outcomes [24]. In a 2015 study [25], decision trees performed slightly better than regression analysis when various age indicators were assessed. Studies using decision trees to estimate age are, however, limited. The advantage of using decision trees is that it provides an easy-to-understand visual representation and a simpler alternative to complex transitional analysis calculations. However, it is important to employ large datasets when using machine learning, as small datasets may result in the model showing a high variance and performing overly optimistic [26].

The aim of this study was to combine dental third molar and anterior inferior apophysis ossification data for purposes of age estimation, by applying a decision tree analysis.

## Materials and methods

### The sample

The sample comprised a total of 871 modern individuals of black South African origin and included 446 males and 425 females with ages ranging from 15 to 24 years (Table 1). The study sample is a subset of individuals analyzed in a previous study that employed traditional statistics [6, 18]. Individuals were included in this study sample if they had both a panoramic and cephalometric radiograph taken on the same day. Data for white individuals used in previously published papers were omitted, as their sample sizes were too small. Individuals of known age, sex and ancestry were selected using a quota sampling method [27]. All individuals were living and analyzed using panoramic and cephalometric radiographs from routine dental treatment at the School of Dentistry, University of Pretoria between 2013 and 2016. Individuals treated at the School of Dentistry encompass all socio-economic groups and thus the sample includes individuals from various backgrounds.

**Table 1:** Age and sex distribution of the total sample

Age (years)	Number of individuals	
	Males	Females
15	29	14
16	47	51
17	56	44
18	52	53
19	46	46
20	58	55
21	56	46
22	50	58
23	38	47
24	14	11
Total	446	425

An additional test sample comprising of 32 individuals (15 males; 17 females) was selected from the School of Dentistry following the same process as the original sample for evaluation of the decision tree model. Their individual ages ranged between 14 and 23 years.

### Scoring of age indicators

The development of the mandibular and maxillary third molars was assessed on panoramic radiographs using a modified version of the original Demirjian 10-stage scoring system [11] to include two additional stages (i.e., F1 and G1) during dental development in the adolescent years [4]. The developmental stages for scoring that were encountered and thus relevant to the years of development (≥ 15 years) in this study are summarized in Table 2.

**Table 2:** Description of scores assigned to the development of the third molar (Demirjian et al. 1973; modified by Solari & Abramovitch 2002)

Stage	Criteria
D	Crown formation has been completed to the dentino-enamel junction and the pulp chamber has a trapezoidal shape
E	Inter-radicular bifurcation formation has begun. Root length is less than the crown length
F	Root length is at least as long as the crown length, with root endings still displaying a funnel shape
F_1_	Root length is about twice the length of the crown, with root endings still displaying a funnel shape
G	Walls of the root canal (radicular pulp) chamber are parallel. The apical foramen remains open
G_1_	Walls of the root canal (radicular pulp) chamber are parallel. Apical foramina not yet fully closed. The periodontal ligament around the apical ending measures ≥ 1 mm
H	Apical foramina are fully closed. The periodontal ligament around the roots is uniform in width

The anterior inferior vertebral ring apophysis development of cervical vertebrae C2, C3, and C4 was assessed on cephalometric radiographs and scored according to a five-stage (0 to 4) scoring system [18]. This scoring system is based on the development of the cervical vertebral ring apophysis from ossification to fusion with the vertebral body as observed on cephalometric radiographs. The stages for scoring are summarized in Table 3.

**Table 3:** Description of scores assigned to the development of the cervical vertebral ring apophysis [18]

Stage	Criteria
0	No ossification of the apophysis visible. The inferior border of the cervical vertebra is flat or show a slight concavity in C2 and C3. The superior border is tapered from posterior to anterior
1	Ossification of the apophysis is evident. No union between the ossification center and the inferior border of the vertebral body has taken place. The apophysis presents as a small radiodense structure
2	The apophysis has begun to fuse with the inferior border of the vertebra at the posterior end of the ossification center. A radiolucent line is visible between the ossification center and anterior aspect of the inferior vertebral body
3	Union has taken place, but a notch is still present between the apophysis and the inferior vertebral body
4	Complete union of the apophysis to the vertebral body indicated by a smooth and intact cortical margin

### Statistical analysis

The data mining software WEKA—Waikato Environment for Knowledge Analysis (version 3.8.5) was used for the construction of a pruned decision tree model. The software was developed by the University of Waikato, New Zealand as a companion to the manual *Data Mining: Practical Machine Learning Tools and Techniques* [28]*.* A ten-fold cross-validation (*k* = 10) was included to prevent overfitting of data.

Male and female datasets were analyzed separately to provide sex-specific decision trees for classification. The known ages of the individuals in the original dataset given as number of years and months were rounded off to the closest whole/natural number for ease of classification. The algorithm was then employed to find patterns in the data that are able to distinguish between the different ages in order to build sex-specific decision trees that may be used for classification.

An estimated age was established for each individual in the test sample (*n* = 32) using the decision trees for males and females, respectively. Actual and predicted ages were compared by means of a Pearson correlation to get an indication of the model’s accuracy and precision.

Intra-and inter-observer repeatability was assessed and reported on during the initial data collection and publication [6, 18] and was not repeated here.

## Results

**Fig. 1:**
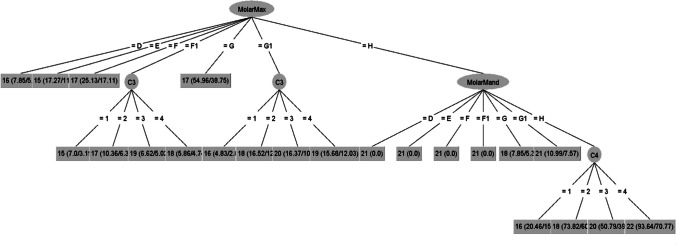
Decision tree for the male dataset (MolarMax = maxillary molar; MolarMand = mandibular molar; C3 = third cervical vertebra ring apophysis; C4 = fourth cervical vertebra ring apophysis)

**Fig. 2:**
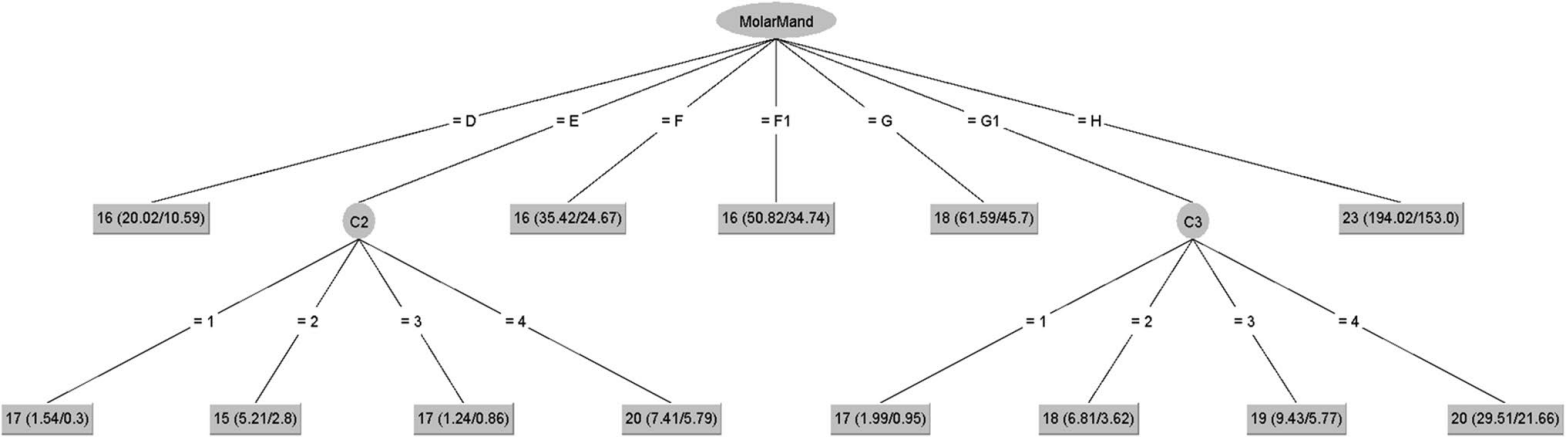
Decision tree for the female dataset (MolarMand = mandibular molar; C2 = second cervical vertebra ring apophysis; C3 = third cervical vertebra ring apophysis)

Sex-specific decision tree models for estimating age were built using the combination of third molar and cervical vertebral ring apophysis age markers. The analysis based on five variables (maxillary third molar, mandibular third molar, C2, C3, and C4 ring apophysis) yielded two decision trees displaying a slightly different pattern between males and females (Figs. 1 and 2). By employing a pruned decision tree algorithm, accuracy of the tree is improved by the removal of variables or nodes that does not significantly contribute to the model. For this reason, not all variables are included in the outcome.

In both the male and female statistical outputs, the prediction accuracy was moderate to low. Classification results for males (446 instances) yielded a 25.5% correctly classified rate with a kappa value of 0.157 (suggesting a fair agreement between observed and expected values). The mean absolute error (MAE) was 0.1704, demonstrating that a moderate to low accuracy and error predicting is present between true and predicted values. The statistical outcome for females performed slightly better, with 28.3% of the 425 instances correctly classified. The kappa value of 0.1836 and MAE of 0.1687 also demonstrated a moderate to low accuracy and error prediction.

The decision tree for males selected both the maxillary and mandibular molars, in combination with C3 and C4 vertebrae (Fig. 1). The root node is indicated as MolarMax (maxillary third molar) and based on the score assigned to its development, the next level of nodes includes C3 (third cervical vertebral ring apophysis) and MolarMand (mandibular third molar). A third and last level is observed incorporating C4 (fourth cervical vertebral ring apophysis) as a terminal node when a score of “H” is assigned to the mandibular third molar development.

The female decision tree model (Fig. 2) found a significant pattern using the mandibular third molar, but not the maxillary third molar, in conjunction with C2 and C3 vertebrae. Only two levels are displayed, with a score of “E” in the mandibular third molar development branching to C2 (second cervical vertebral ring apophysis) and a score of “G1” branching to C3 (third cervical vertebral ring apophysis).

The sequence of variables presented by both trees suggests that third molar development, whether mandibular or maxillary, is likely to be the most reliable and accurate variable. When evaluating the trees, it was evident that all variables studied and incorporated into the analysis featured at some point, albeit not in both male and female classifications.

**Fig. 3:**
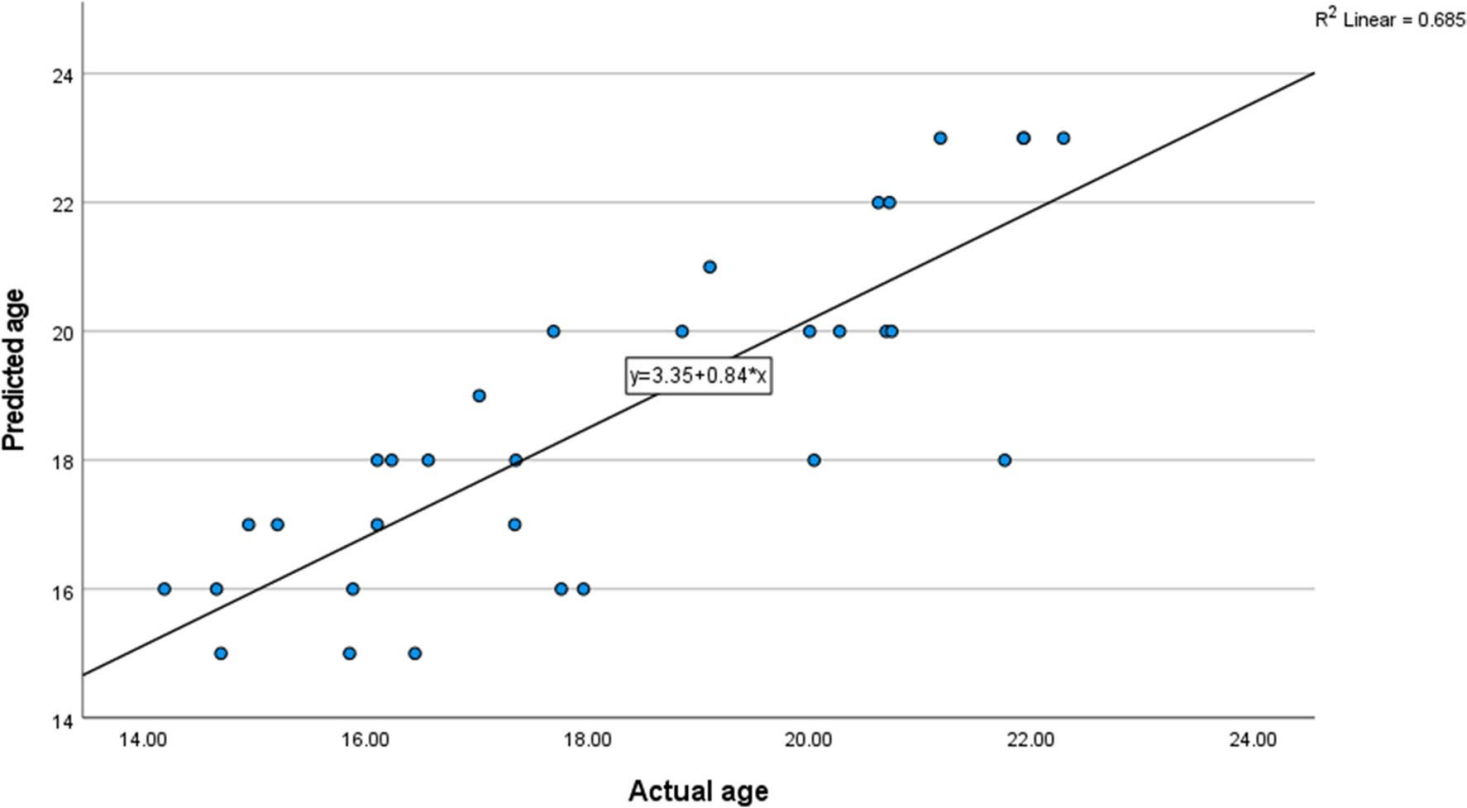
Linear relationship between actual and predicted ages for the test sample

The outcome of the test sample is summarized in Table 4. The results from the test sample showed that the actual ages (in years and months) were closely related to the predicted ages (in years only), with an average of 0.47 years age difference between the values. The maximum age difference between the true and predicted age was -3.76 years (minus indicating a predicted age younger than the actual age), while the minimum age difference was 0 years. The value of -3.76 was due to one outlier individual whereas if this individual was excluded, the ranges would be between -2.04 to 2.31. However, as far as could be established, the age of this individual was correct and it should be considered that such outliers will potentially be encountered in these analyses. A Pearson’s correlation with the best fit linear relationship (*r* = 0.828, *R*2 = 0.685) indicated a high positive relationship between the true and predicted ages (Fig. 3). This suggests that there is a strong relationship between the true and estimated ages, with an average deviation from the true age of less than 6 months. However, the method does seem to overage, rather than underage individuals.

**Table 4:** Test sample results showing the true age, sex, estimated age (decision tree), and the difference between the actual and estimated age

Individual #	Sex	True age	Estimated age	Age difference
1	F	14,65	16,00	1,35
2	F	15,88	16,00	0,12
3	F	16,1	18,00	1,9
4	F	16,23	18,00	1,77
5	F	16,56	18,00	1,44
6	F	17,02	19,00	1,98
7	F	17,35	18,00	0,65
8	F	17,76	16,00	-1,76
9	F	17,96	16,00	-1,96
10	F	18,85	20,00	1,15
11	F	20,27	20,00	-0,27
12	F	20,69	20,00	-0,69
13	F	21,18	23,00	1,82
14	F	21,76	18,00	-3,76
15	F	21,93	23,00	1,07
16	F	21,93	23,00	1,07
17	F	22,29	23,00	0,71
18	M	14,18	16,00	1,82
19	M	14,69	15,00	0,31
20	M	14,94	17,00	2,06
21	M	15,2	17,00	1,8
22	M	15,85	15,00	-0,85
23	M	16,1	17,00	0,9
24	M	16,44	15,00	-1,44
25	M	17,34	17,00	-0,34
26	M	17,69	20,00	2,31
27	M	19,1	21,00	1,9
28	M	20,00	20,00	0
29	M	20,04	18,00	-2,04
30	M	20,62	22,00	1,38
31	M	20,72	22,00	1,28
32	M	20,74	20,00	-0,74

**Fig. 4:**
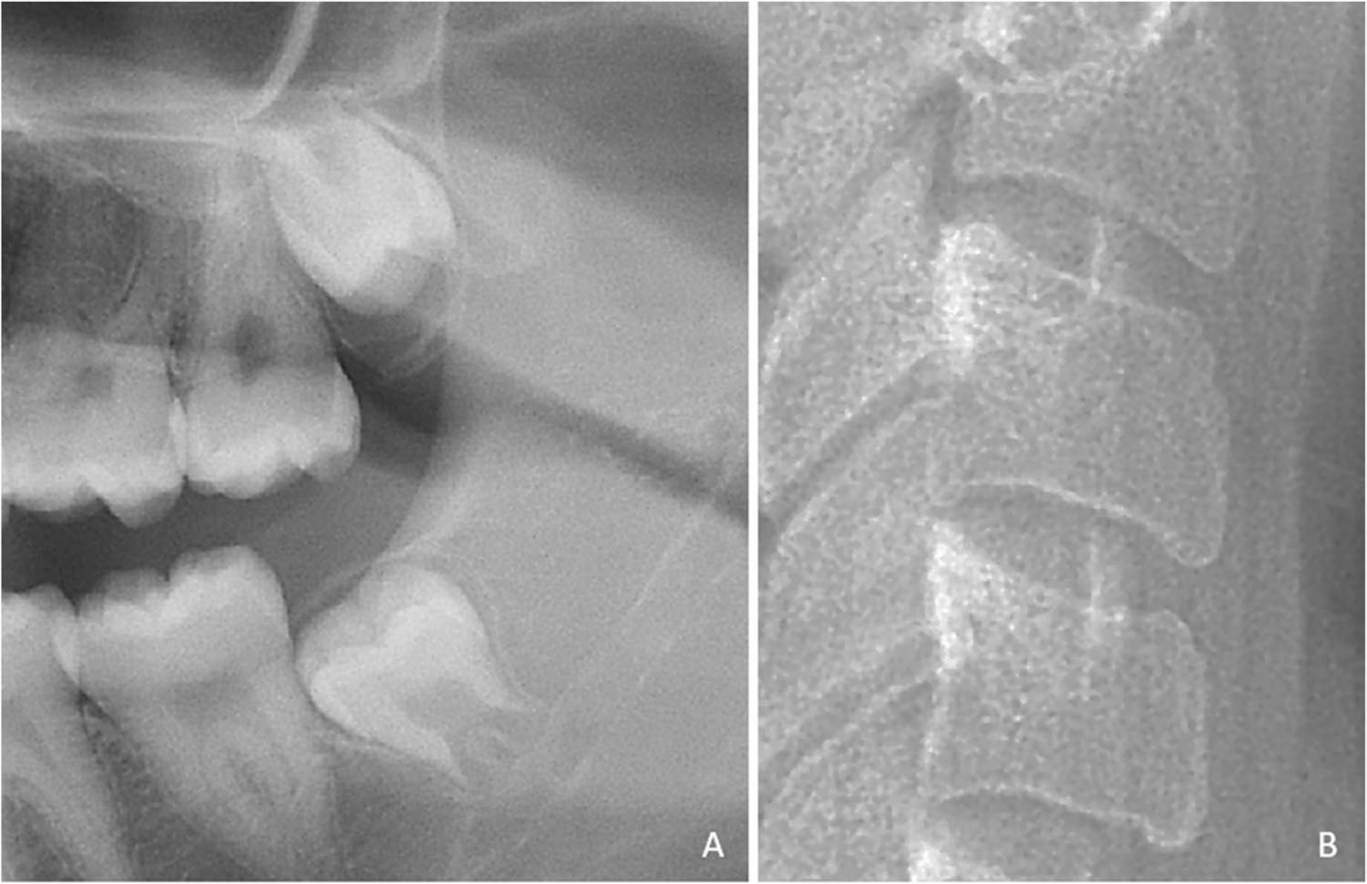
Example of a female individual with chronological age 17.76 years. The cropped panoramic radiograph (**A**) shows the left third molars and the cropped cephalometric radiograph (**B**) shows the inferior surfaces of cervical vertebrae C2, C3, and C4. The maxillary third molar was classified as stage E and the mandibular third molar was classified as stage F. The anterior inferior apophysis ossification stages of cervical vertebrae C2, C3, and C4 were all classified as stage 4

**Fig. 5:**
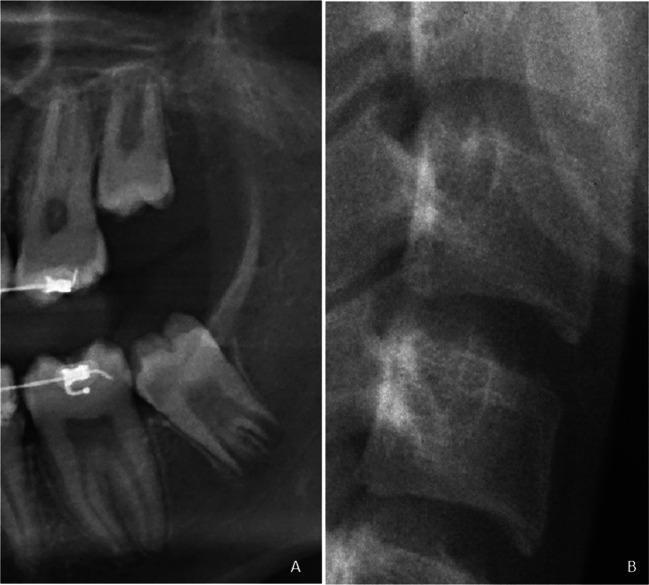
Example of a male individual with chronological age 17.34 years. The cropped panoramic radiograph (**A**) shows the left third molars and the cropped cephalometric radiograph (**B**) shows the inferior surfaces of cervical vertebrae C2, C3, and C4. The maxillary third molar was classified as stage F1 and the mandibular third molar was classified as stage F1. The anterior inferior apophysis ossification stages of cervical vertebrae C2, C3, and C4 were classified as stage 1,2,2 respectively

To demonstrate how to use the decision trees, two examples were done based on cases shown in Figs. 4 and 5. The radiographic images of a female individual with a true age of 17.76 years is shown in Fig. 4. The maxillary molar was scored as stage E; the mandibular molar was scored as stage F; C2, C3 and C4 were all scored as stage 4. The female decision tree dataset was used to calculate the age estimate (Fig. 2). The mandibular molar (stage F) gives a direct age estimate of 16 years.

The radiographic images of a male individual with a true age of 17.34 years is shown in Fig. 5. The maxillary molar was scored as stage F1; the mandibular molar was scored as stage F1; C2, C3 and C4 were scored as stage 1,2,2 respectively. The male decision tree dataset was used to calculate the age estimate (Fig. 1). The maxillary molar (stage F1) follow the path to C3. From C3 the stage was scored as stage 2 which gives an age estimate of 17 years.

## Discussion

After the age of 14 years, estimating the age in living individuals becomes challenging, as there are only a limited number of age indicators available [29]. Because the outcomes of such estimations have significant implications, there is a need for accurate and reliable methods in this context [30]. These methods should be practical, accurate, and user-friendly. Approaches that fulfil these criteria are essential. Some researchers have proposed combining dental observations, like third molar development, with age-related skeletal indicators to minimize the wide age estimation prediction intervals [31, 32], which is the approach we followed here. Intra-and inter-observer repeatability was assessed and reported on during the initial data collection and publication [6, 18]. The intra-observer repeatability showed substantial agreement for scoring mandibular and maxillary teeth (Cohen’s kappa values: 0.8511 and 0.9263, respectively), while inter-observer repeatability indicated substantial agreement for maxillary third molars (Cohen’s kappa value: 0.6287) and moderate agreement for mandibular third molars (Cohen’s kappa value: 0.5107) [6]. Assessment of the anterior cervical vertebral ring apophysis ossification stages findings on inter-observer repeatability suggested that the level of agreement among raters surpassed what would be anticipated if all raters assigned their ratings entirely at random, according to Fleiss’s method [18].

The advantage of using a decision tree is that it is simple and easy to use when attempting to classify individuals either as younger or older than eighteen years of age. The decision tree can easily be visualized and is easy to understand and to interpret the data. Weak correlations are excluded to increase the accuracy of the model. It is advised that large training samples should be used, as larger samples would produce more reliable models. Langley et al. [33] reported a correct sex classification rate of 93.5% using a decision tree with a training sample of 209 individuals. Studies using a smaller sample size may achieve higher prediction accuracy but with a reduced applicability to diverse samples. Conversely, studies using a larger sample size possess greater generalisation potential but may sacrifice precision [34, 35]. In summary, when comparing machine learning study outcomes, it is essential to consider sample sizes. To evaluate the model applicability, it is recommended to validate prediction models in independent samples through direct application. It is also advisable that the outcomes should be tested by a large test sample. However, as proof of concept, this seems to be a promising approach going forward. Unfortunately, at the moment, only a point estimate is available and no confidence interval or age range is provided.

Regarding the eruption and mineralization of third molars, it has been observed that black African individuals exhibit accelerated development compared to individuals from European ancestry, while Asians tend to show relative retardation in their development [36]. Therefore, to assess the development of third molars accurately in age estimation, it is essential to utilize population-specific reference studies [37]. Age estimations based on the development of the third molar can be prone to error due to the high variability of this tooth [38]. The algorithm identifies variables that make a minimal contribution and subsequently omits them from the corresponding decision tree. In our male decision tree both the maxillary and mandibular third molar were included but in the female decision tree the maxillary third molar was omitted. The C2 ring apophysis was also excluded in males, while in the female dataset the C4 ring apophysis and the maxillary M3 were excluded.

In our study, the average difference between the chronological age and the estimated age using decision trees was less than 6 months for the entire test sample, which is a very good outcome. For all the females in the test sample, on average, the difference was less than 5 months and for the males less than 7 months. However, on the individual level, several individuals were vastly over- or under-aged, demonstrating the difficulties with age estimation due to differences in individual maturation. It is imperative to consistently apply the minimum-age concept in age estimation methods to ensure that the forensic age estimate assigned to the evaluated individual remains consistently below their actual age, preventing overestimation [39]. The decision tree for females up to the age of 17.35 years frequently overestimated their age, particularly classifying minors as adults. Similarly, the decision tree for younger males also tended to overestimate age, with fewer instances of classifying minor males as adults. Overestimation poses challenges, especially when individuals under 18 years old are inaccurately categorized as over eighteen, as observed in cases such as #3, #4, #5, #6, and #7 for females and #26 for males (Table 4). A larger test sample is needed to specifically assess what is happening around the ages of 16 to 18 years, where overestimation can have serious consequences for the individual. Combing different age estimation features may also yield different results when using decision trees. An interesting observation was that the decision tree algorithm showed that males (Fig. 1) with a MolarMax stage E are indicated as younger than those with stage D. Similarly, in females (Fig. 2) a MolarMand stage D was older than a stage E individual with a stage 2 anterior C2 ring apophysis. A possible explanation for this could be the small differences between subsequent stages as well as individual variation within the sample related to these ages.

All the information needed to classify the different development features can be obtained from radiographs as recommended by the European Asylum Support Office (EASO) practical guide on age assessment [40]. The intra- and inter-observer agreement from the original studies indicated substantial agreement for scoring third molar as well as cervical vertebral ring apophysis development [6, 18]. The utilization of both panoramic and cephalometric radiographs for age estimation yields a positive result while minimizing radiation exposure to the person. However, it is essential to adhere to the radiation protection guidelines suggested by the International Commission on Radiological Protection (ICRP) for younger individuals [41].

## Conclusion

Machine-learning and artificial intelligence provides exciting new opportunities when it comes to age estimation, although more research is needed. Machine learning requires large samples, something that is not always available. The authors recommend using a decision tree in conjunction with other methods, despite the relatively narrow margin of error. As in any case of age estimation in living individuals, the characteristics of the individual, including his/her growth and nutritional status should be considered, and it is unlikely that a single solution to this problem will ever be obtained. Nevertheless, the decisions trees demonstrated here provides a valuable addition to the armamentarium that is available for practitioners when it comes to age estimation around the critical age of 18 years.

## Data Availability

The data that support the findings of this study are available on request from the corresponding author, (AU).
